# Practical realization of the watt from Planck’s constant using radiation pressure

**DOI:** 10.1088/1681-7575/ad844b

**Published:** 2024

**Authors:** Brian J Simonds, Kyle A Rogers, Sven Schulze, David Newell, Gordon Shaw, Johannes Wahl, Paul A Williams, John H Lehman

**Affiliations:** 1 Sources and Detectors Group, National Institute of Standards and Technology, Boulder, CO, United States of America; 2 Mass and Force Group, National Institute of Standards and Technology, Gaithersburg, MD, United States of America; 3 Institut für Strahlwerkzeuge (IFSW), University of Stuttgart, Stuttgart, Germany

**Keywords:** radiometry, radiation pressure, high-power laser, electrostatic force balance

## Abstract

A primary force standard is implemented to realize the watt through Planck’s constant by means of radiation pressure at the kilowatt level. The high amplification laser-pressure optic, or HALO, is a multiple reflection radiation pressure apparatus used for absolute radiometry of high-power lasers. In this work, a primary standard electrostatic force balance is used to measure the reflection-enhanced optical forces. With this configuration, the HALO is used to measure laser powers in the range of 100 W–5000 W from a 1070 nm fiber laser. The expanded uncertainty of the 5 kW measurement is 0.12%, which is both the lowest uncertainty multi-kW measurement and radiation pressure-based measurement to-date. The HALO result was validated against a thermal primary standard using a calibrated transfer standard at 2 kW. The degree of equivalence was 0.78% ± 1.12%, which demonstrates agreement within the uncertainties of these two primary standards.

## 1. Introduction

Prior to the 2019 re-definition of the SI, it was recognized that the watt could be realized using Planck’s constant through the measurement of force (or the kilogram) [1]. By using radiation pressure (RP) to convert photon momentum into a mechanical force, the watt can be expressed in terms of Planck’s constant and the hyperfine splitting of ^133^Cs [2]. Therefore, a practical realization of the watt using RP requires a primary force standard with traceability to these same constants without relying on an intermediary measurement. Such an experiment was carried out at the 1 W level but with an expanded uncertainty of around 5% [3]. In the range of 1 W–10 W, Vasilyan *et al* worked towards a practical realization but due to an unexplainable systematic discrepancy were unable to ascribe an expanded uncertainty [4]. At the kilowatt level, previous efforts by our group achieved low combined expanded uncertainty (0.26% at 10 kW, *k* = 2), but it was not a realization because a mass artifact was needed for calibration [5]. In this work, we demonstrate a practical realization of the watt using RP by employing a primary force standard electrostatic force balance with direct traceability to Planck’s constant.

**Figure 1.**
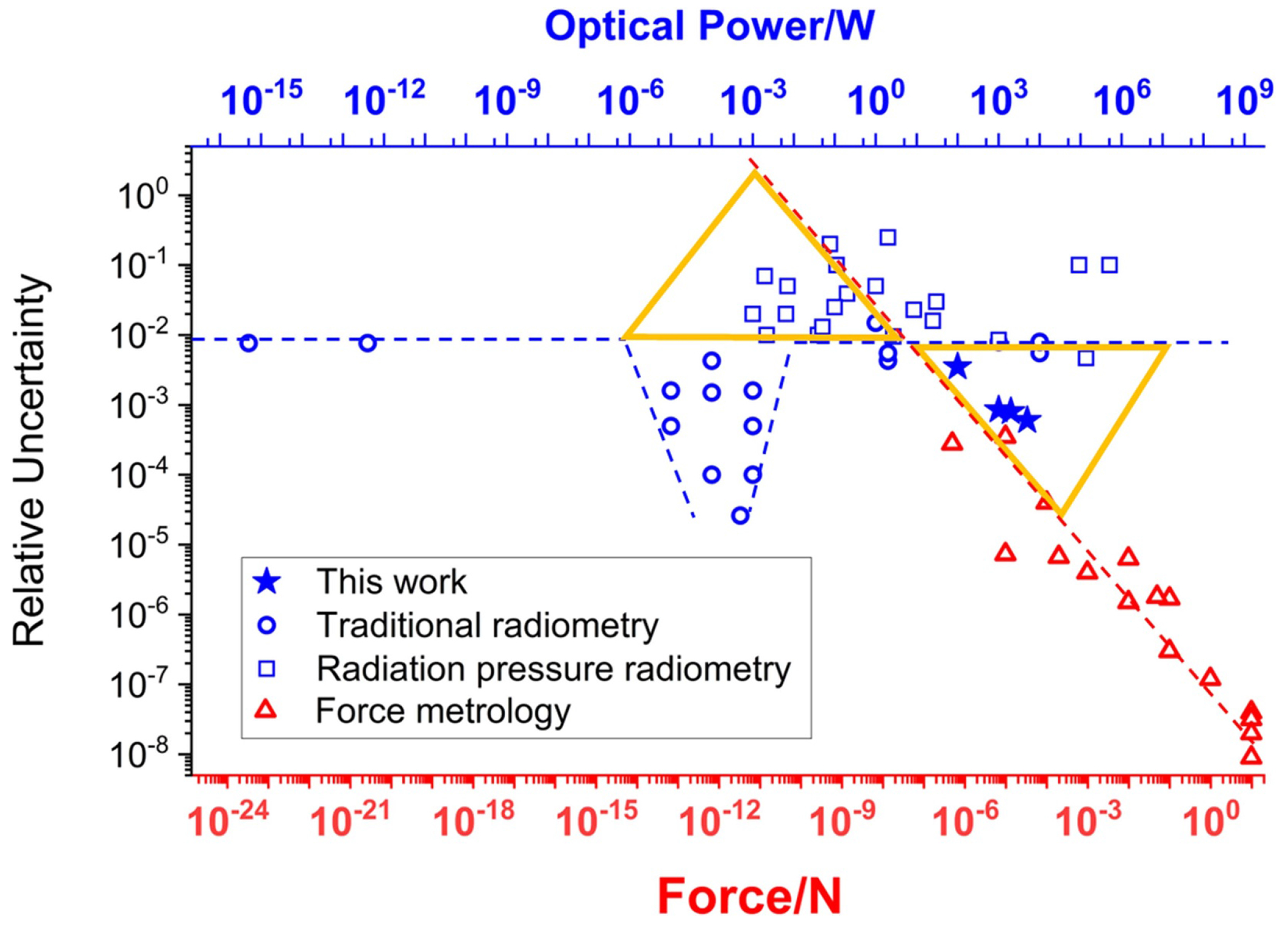
The ‘bow tie’ plot showing measurement combined standard relative uncertainty of published measurements of optical power (upper axis, blue) and object weight expressed as force (lower axes, red). The scaling between the upper and lower axes is defined by equation (1) for an incidence angle *θ* = 0. Power measurements were performed by traditional (thermal or diode-based) means [2, 10], as well as single [3, 11–23] and multiple reflection (this work) radiation pressure techniques. The dashed lines are included to guide the eye to indicate where assessing a force from its equivalent optical power, or vice versa, could yield lower uncertainties than traditional approaches (yellow triangular regions).

Optical power measurements have traditionally been performed thermally by measuring heat converted from radiant energy in some form or another. This has been true at low powers using photon counting [6] or at high continuous wave powers above 100 kW [7–9]. Figure 1 plots the relative uncertainty of published traditional (thermal or diode based) optical power measurements across 21 orders of magnitude (blue circles referenced to the top ordinate) [2, 10]. This non-exhaustive list of references represents the state-of-the-art. Relative uncertainties are roughly constant at around 1% across all power ranges except the μW to mW range (blue dashed line). The region of lower uncertainties in the μW to mW range is due to substantial effort over several decades by the radiometry community in developing the cryogenic radiometer [10].

Here we demonstrate that it is possible to achieve low uncertainty optical power measurements with a primary force balance (in air) and without requiring traceability through mass artifacts. Radiation pressure has been experimentally recognized as a means to convert light into a mechanical force for over 100 years [11, 12] and is now recognized as due to the transfer of photon momentum. RP-based optical power measurements and their published uncertainties have been included in figure 1 as squares, the values of which also correspond to the top axis [3, 11–23]. In general, their uncertainties are at or above the 1% level established for most other traditional optical power measurements. Overlaid on figure 1 are the published standard combined uncertainties of force measurements (red triangles, bottom axis) realized using physical objects through subdivision of the kilogram artifact [24, 25], electromagnetic force balances (Kibble–like) [26–28], and electrostatic force balances [24]. These results show a linearly decreasing uncertainty with increasing force (red dashed line) revealing a universal truth about force metrology: larger forces can be measured with higher accuracy. The top and bottom horizontal axes are aligned according to the relationship between the RP force of light incident on a mirror as

(1) $$P=\left(\frac{c}{2R\;\cos\theta }\right)\;F\;$$

where *c* is the speed of light, *R* is due to mirror reflectance and absorption, and *θ* is the angle of incidence. In order to equate the top and bottom axes in figure 1, we assume perfect mirrors (*R* = 1) and normal incidence (*θ* = 0) in equation (1). The crossover in uncertainty between traditional optical power meters and force measurements occurs at approximately 5 W. Therefore, above 5 W it should be advantageous to realize optical power using RP by leveraging the improved accuracy of force metrology. Below 5 W, or equivalently about 30 nN, the accuracy of small force metrology can be improved by using optical power techniques. These two regions have been highlighted on figure 1 with yellow triangles, giving the appearance of a bow tie. The upper left leaf of the bow tie shows that some RP measurements have achieved lower uncertainties than predicted by traditional force metrology projections. However, the lower right leaf (high optical powers) remains largely empty except for the work discussed in this manuscript.

Most of the RP experiments shown in figure 1 are from single reflection devices where incident laser light is reflected once from a mirror attached to some sort of force transducer. Such a device has been developed by the National Institute of Standards and Technology (NIST) for high laser powers and has thus far been demonstrated up to 140 kW [2, 22, 23, 29–33]. Although the forces involved at these large powers would suggest that lower uncertainties should be possible from a force metrology perspective, their accuracy remains at roughly 1%. This is primarily due to the fact that the conditions under which these large laser measurements are performed are significantly less stable than laboratory environments for precision force metrology [21].

The RP force applied can be further increased by reflecting the light back onto the mirror attached to the force transducer. If the angle of incidence remains the same and there is negligible loss along the beam path, then the force is increased linearly by the number of reflections on the mirror. This approach has been pursued by NIST for high-power (>1 kW) laser measurements using a device called the HALO, or high amplification laser-pressure optic, which produced an expanded uncertainty (95% confidence interval) of 0.26% at 10 kW [5]. At lower powers (<10 W), others have investigated similar techniques albeit with uncertainties much larger than 1% [3, 4, 34].

This work extends from previous HALO work in three main regards. The first is that the commercial force balance used in [5] is replaced by a custom-built primary standard electrostatic force balance (EFB) [35]. This means that a mass artifact is no longer necessary for realizing force, and that these laser power measurements have direct traceability to Planck’s constant. Second, we report an improvement in the HALO uncertainty of roughly a factor of two by improving alignment and reducing statistical noise. Third, we present results comparing the HALO result to our thermal primary optical power standard that shows agreement within the uncertainty of the comparison measurement.

## 2. Methods

**Figure 2.**
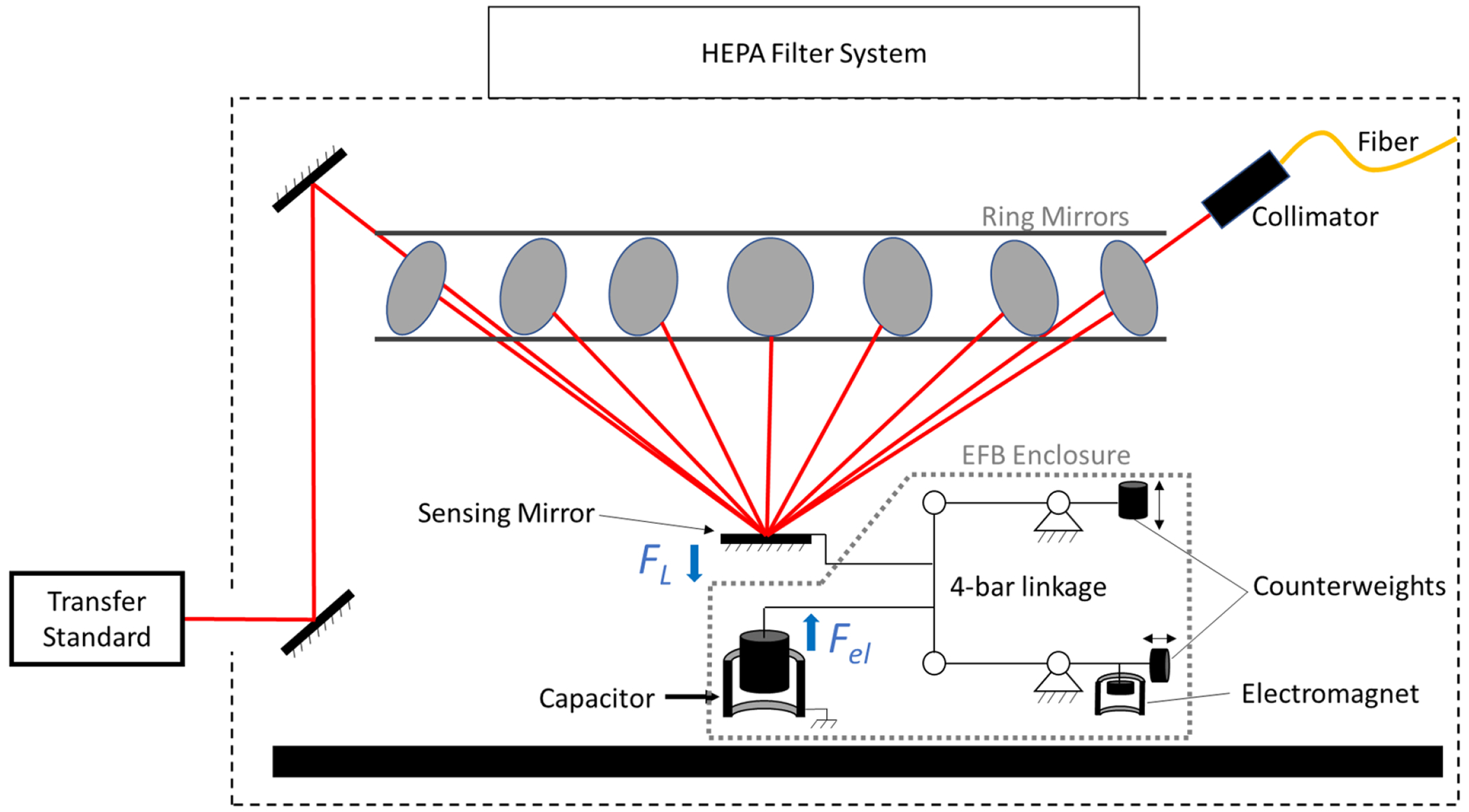
A diagram of the HALO experiment. The red lines indicate the approximate beam path. The dashed line shows the extent of the enclosure that houses the experiment is approximately 1.5 m wide by 1 m high.

The HALO design is fully explained in [5] and will be briefly described here. Laser light is reflected onto a sensing mirror 14 times by 13 ring mirrors arranged in a plane above the sensing mirror (figure 2). The ring mirrors are arranged such that the angle of incidence on the sensing mirror of each pass of the laser is 44.8°. As a result, the force gain factor is equal to the number of reflections. After the multiple reflections on the sensing mirror, the beam enters a periscope before exiting the HALO enclosure. The total beam path is approximately 12 m before being captured by either a beam dump or a calibrated thermopile transfer standard used for comparing HALO to a thermal primary optical power standard.

The laser used is a commercial multimode, continuous-wave 1070 nm fiber laser. It has a stable output power range of approximately 70 W–10 000 W. A low-power visible (red) guide laser can propagate colinearly with the high-powered beam and is used for aligning the ring mirrors. This is performed by replacing the first ring mirror with an alignment target that has concentric rings spaced 2 mm apart. The position and angle of the laser collimator is then adjusted until the guide beam is centered on the target. The alignment target is moved to the next ring mirror in the optical path with the first ring mirror returned to its mount. The angle of the first ring mirror is now adjusted until the guide beam is centered on the target in the second ring mirror position. This process is iterated until all the ring mirrors are aligned. Using this alignment method, we conservatively estimate that the beam is centered on the ring mirrors to within 1 mm. This, along with the as-built geometry of the system is used in a Monte Carlo simulation [5] to estimate the measurement uncertainty contribution due to optical alignment.

In order to eliminate light lost to scattering by airborne particles, HALO is kept in a clean enclosure with positive pressure provided by a high efficiency particulate air (HEPA) filter. A recirculating HEPA filter and air deionizer are also used to reduce particulate contamination. These systems are only in use between optical power measurements since their mechanical vibration and air currents would disrupt the sensitive force measurements. We have estimated the potential loss of light due to scattering by combining Mie theory with absolute measurements of airborne particles in the HALO enclosure. Assuming a complex index of refraction of 1.5 + 0.01*i* of the particles, we calculate a relative total extinction (absorption plus scattering) loss of 2 × 10^−7^ with particle counts measured one hour after the HEPA fan has been switched off. Particle counts one week after the HEPA fan had been off only increases this value to 1 × 10^−6^. These levels are negligibly small compared to the uncertainty of our measurement.

### 2.1. Electrostatic force balance design

The RP force scales linearly with applied optical power. So, for HALO to operate between 0.1 kW and 10 kW, the balance will need to record forces between 6.6 μN and 660 μN. A single reflection at 44.8° angle of incidence of 1 kW laser light delivers 4.7 μN of force. After HALO’s 14 reflections, this becomes 66 μN, which is the weight of a 6.7 mg mass in our laboratory. In this range of forces, there are two types of primary force standards: the electromagnetic force balance, commonly referred to as a Kibble balance [28], and the electrostatic force balance (EFB). We chose an EFB for this work due to its lower achievable uncertainty in the range of forces required by HALO [24].

Figure 2 shows a schematic of the EFB specifically designed for use in HALO. The sensing mirror feels the full multiplied force of the applied laser beam. This force is translated to the inner electrode of a cylindrical capacitor through dual four-bar linkages placed side-by-side. This design ensures that the sensing mirror has only vertical translation, minimizing rotation and therefore corner loading error [35]. The RP force, *F_L_*, on the sensing mirror is balanced by an electrostatic force, *F_el_*, provided by the capacitor according to

(2) $$F_{L}=-F_{el}=-\frac{1}{2}\frac{\text{d}C}{\text{d}x}V^{2}$$

where *V* is the applied voltage. An interferometer (not shown in figure 2) aligned to the axis of local gravity measures displacement of the capacitor inner electrode to feedback control the applied voltage thereby maintaining the sensing mirror at a null position. Counterweights on the four-bar linkage are used to control damping and stiffness. The stiffness of the mechanism is kept low (<0.1 N m^−1^) as force noise increases with stiffness.

**Figure 3.**
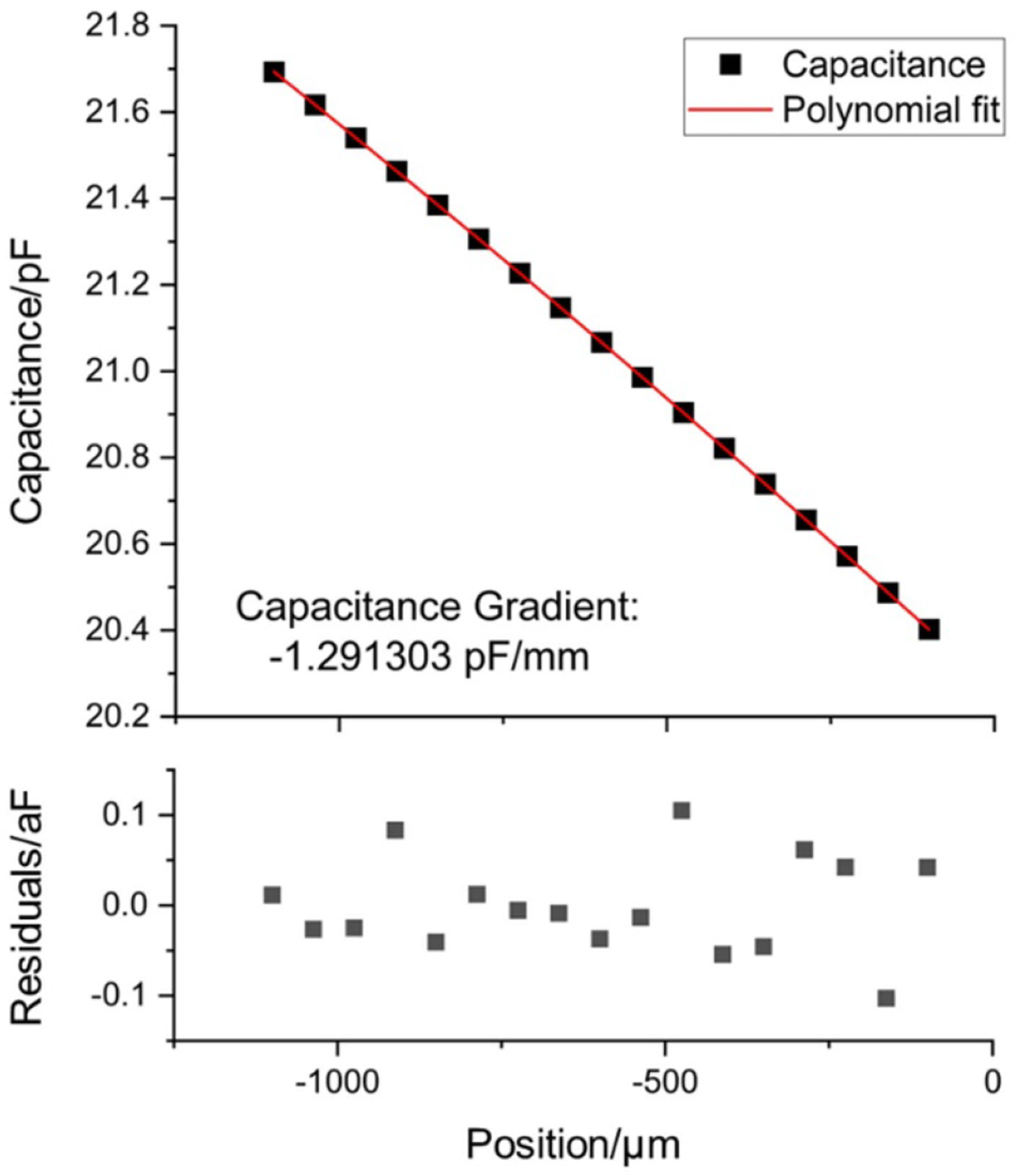
A representative EFB capacitance gradient measurement and fit result.

The capacitance gradient, d*C*/ d*x*, is determined by a separate experiment using the force coil and the interferometer [36]. A typical curve for a single sweep of capacitance versus sensing mirror position is shown in figure 3. The curve is fit to a fourth order polynomial from which a gradient is calculated. The lower plot shows the residuals of this fit, which are five orders of magnitude lower than the measured capacitance. A single capacitance gradient value is determined from the average of several sweeps across position, typically 8, with the standard deviation of these measurements on the order of 0.01%. Capacitance gradients are measured before and after the series of laser power measurements (either 100 or 200 laser pulses) to account for any potential drift in its value. Pre and post measurement gradients are averaged together with their small standard deviation used as the uncertainty shown in table 1. Included in this value are the effects of temperature on the capacitance over the timescale of the measurement. Voltage was measured using a voltmeter calibrated in the same manner as previous work [24] and is traceable to a NIST Josephson standard. Capacitance was measured using a high-precision capacitance bridge calibrated by the vendor to be traceable to a NIST quantum Hall system through an AC/DC impedance transfer to the calculable capacitor. The interferometer uses an integrated acetylene gas cell to maintain a set frequency ratio to the hyperfine splitting frequency of ^133^Cs [37]. Because the electrical and optical measurements used in the EFB are all traceable to fundamental constants, the watt is therefore also realized within the SI as described in the appendix of [2].

### 2.2. Design of experiment

Measurements were made with the HALO at 100 W, 1 kW, 2 kW, and 5 kW of nominal incident laser power. Laser exposures lasted 30 s with 10 additional seconds of power ramping on and off with 60 s of settling time (laser off) between. Each measurement is the average result of 100 exposures, except for the 100 W measurement which was 200.

HALO results were validated against the NIST high-power primary standard calorimeter [38] using a calibrated transfer standard placed at the exit of the HALO enclosure (figure 2). Although this configuration was used to compare two primary optical power standards, it also demonstrates the unique ability of a RP-based optical power sensor to simultaneously measure the laser power while also delivering the light to a device-under-test. This effectively creates a ‘calibrated laser source.’

## 3. Results and discussion

Table 1 gives the complete uncertainty budget for the measurements discussed here. The optical alignment uncertainty is the cumulative effect of the potential variations in incident angle on the sensing mirror. Its contribution to laser power uncertainty is determined using a Monte Carlo simulation to compute the effect of the beams being imperfectly centered on the ring mirrors [5]. The ‘collimator’ uncertainty results from potential chromatic effects due to the difference in the visible alignment beam and the infrared high-power beam.

Noise uncertainty is the statistical variation of a set of measurements and is defined as the standard deviation of the mean (SDOM) of all measurements in a series. As seen in table 1, this component decreases as the applied laser power increases. This is expected as larger applied forces have improved signal-to-noise (see figure 1). For laser powers of 2 kW and below, this is the dominant source of uncertainty whereas the 5 kW measurement is limited by alignment uncertainty. Therefore, the precision of the lower power measurements can be improved by increasing the number of measurements whereas at 5 kW and above it cannot.

In previous work, the capacitance gradient of another similar balance was found to vary with temerpature at the level of 4.2 × 10^−15^ F m^−1^ per K [5]. No such systematic thermal drift was observed with the EFB measurements, indicating the effect is within the uncertainty of the current capacitance gradient uncertainty. We also estimated the potential thermal effects of optical power absorption by the sensing mirror by assuming that the 0.001% of light not reflected was absorbed. Even at 5 kW, we estimated that this small amount of absorbed power would not appreciably distort the mirror coating and affect its reflectivity.

In precision capacitance measurements, a surface potential can arise due to a combination of a contact potential and possible contaminants adsorbed on the electrodes. The relative effect of the surface potential is described in [24]. We estimate the surface potential to be within a range of ±20 mV from measurements of Cu surfaces similar to that of our Cu capacitor [39]. The effect of the surface potential is treated as an uncertainty and is given in table 1. It is at least an order of magnitude less than the dominant component at all power levels, which means polarity reversal is not required to compensate for the surface potential at this uncertainty level.

Uncertainty components related to the EFB not already discussed (corner loading, stray capacitance, capacitor alignment, capacitor motion, and transfer of length) are discussed in detail in previous publications [24, 35, 36] and are significantly smaller than the dominant uncertainties for the laser power measurements. Material hysteresis effects of the balance mechanism were not included as previous measurements determined them to on the order of ten parts per trillion [24].

**Figure 4.**
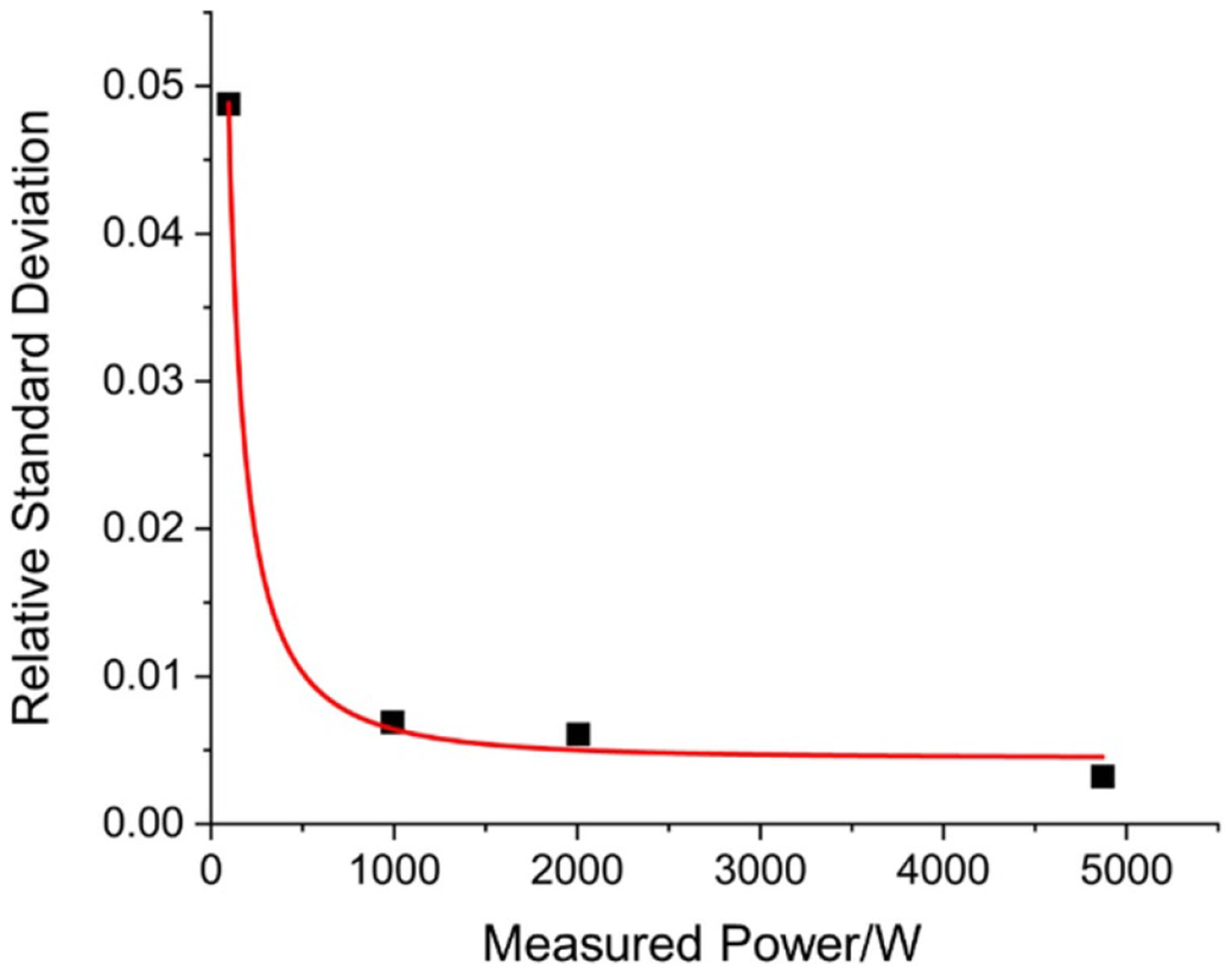
The relative standard deviation versus the measured laser power. The fit is to equation (3), which separates the statistical noise into power-independent ($\sigma_{P}=4.6\;W$) and power-dependent ($\gamma_{T}=0.004$) components.

The relative standard deviation for measured applied laser powers are shown in figure 4. These values are the standard deviation of all measurements at a particular laser power, divided by the power measured. They differ from the noise uncertainty component in table 1 by the square root of the number of measurements. As is common with radiation pressure-based measurements, the standard deviation decreases rapidly with increased laser power [30]. The statistical uncertainty can be separated into power-independent background noise $\sigma_{P}$ and a power-dependent drift non-linearity term $\gamma_{T}$ [22, 30]:

(3) $$u_{\text{sys}}=\sqrt{{\left(\frac{\sigma_{P}}{P}\right)}^{2}+\gamma_{T}^{2}}$$

Equation (3) is used to fit the relative standard deviations in figure 4 where it is found that the power-independent noise, $\sigma_{P}$, is 4.6 W and the power-independent term, $\gamma_{T}$ is 0.004.

### 3.1. Lowest uncertainty multi-kW measurement

**Figure 5.**
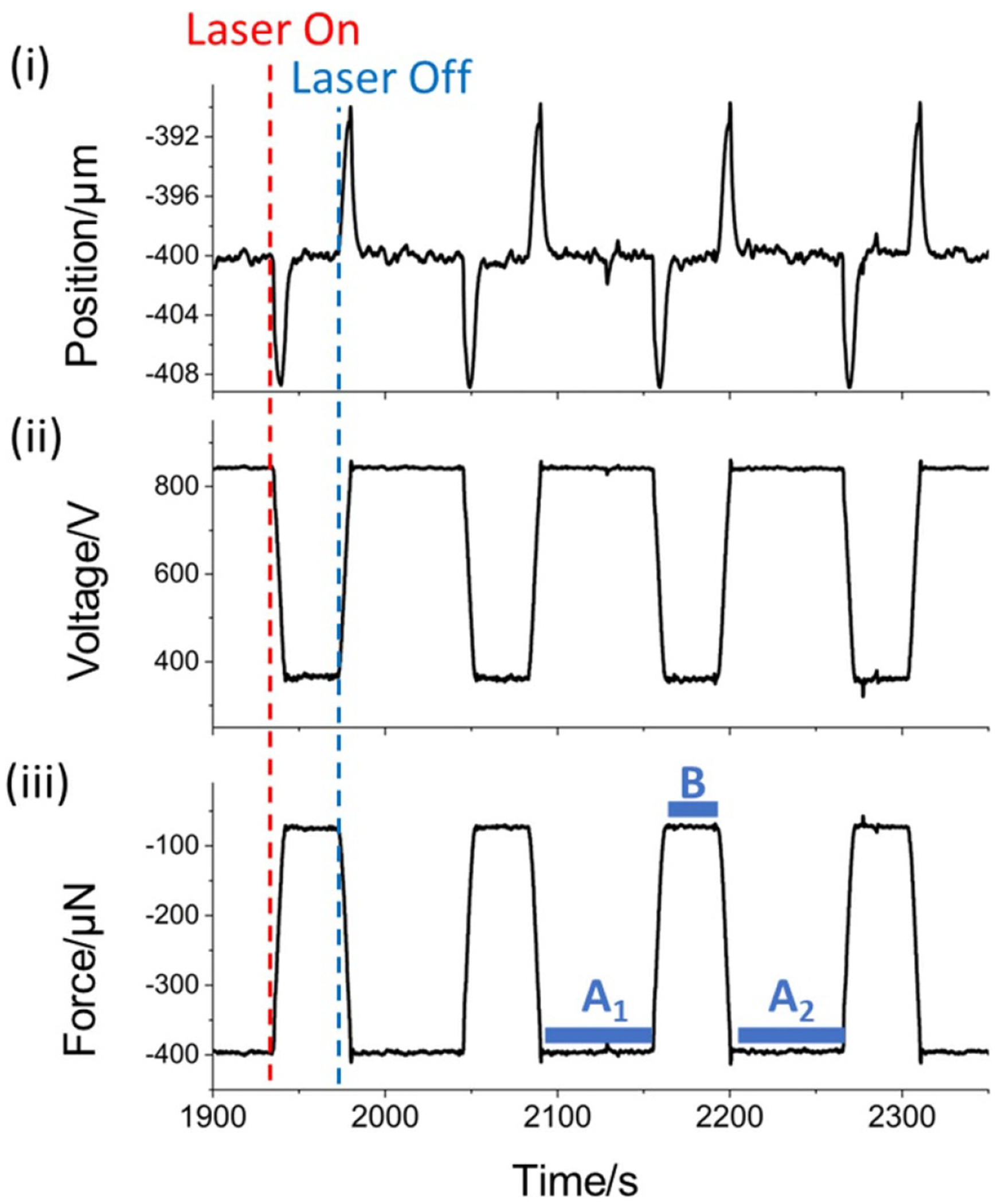
A limited time series of the 5 kW measurements that show (i) the sensing mirror position, (ii) the voltage applied to the EFB, and (iii) the measured force on the EFB. The blue regions of (iii) (A_1,2_ and B) mark the regions used in the ABA analysis.

The measurements at 5 kW have an uncertainty of 0.12%, which is the most accurate multi-kW optical power measurement to-date and the most accurate RP-based measurement at any power. This measurement was the result of 100 repeated laser pulses and figure 5 shows a time series of several of these pulses. The upper plot (figure 5(i)) shows the recorded position of the sensing mirror measured with the interferometer. Brief downward and upward spikes occur when the laser is turned on and off, respectively. These regions are excluded from the data analysis. A 10-second power ramp is applied to the beginning and end of the laser pulse to minimize the impulse felt by the balance. The feedback control of the EFB adjusts the voltage across the cylindrical capacitor (figure 5(ii)) and maintains the original position of the sensing mirror to within 1 μm during steady-state operation. The voltage is then converted to a force using equation (2), which is shown in figure 5(iii). An ABA method is used for analyzing the computed force for each laser pulse, which effectively eliminates drift on the timescale of individual pulses [40]. By this method, the force of each laser pulse $\left(F_{\mathrm{pulse}}\right)$ is computed by subtracting the mean force for 60 s before $\left(A_{1}\right)$ and after $\left(A_{2}\right)$ from the mean force when the laser has a constant output (*B*):

(4) $$F_{pulse}=B-\frac{A_{1}+A_{2}}{2}$$

**Figure 6.**
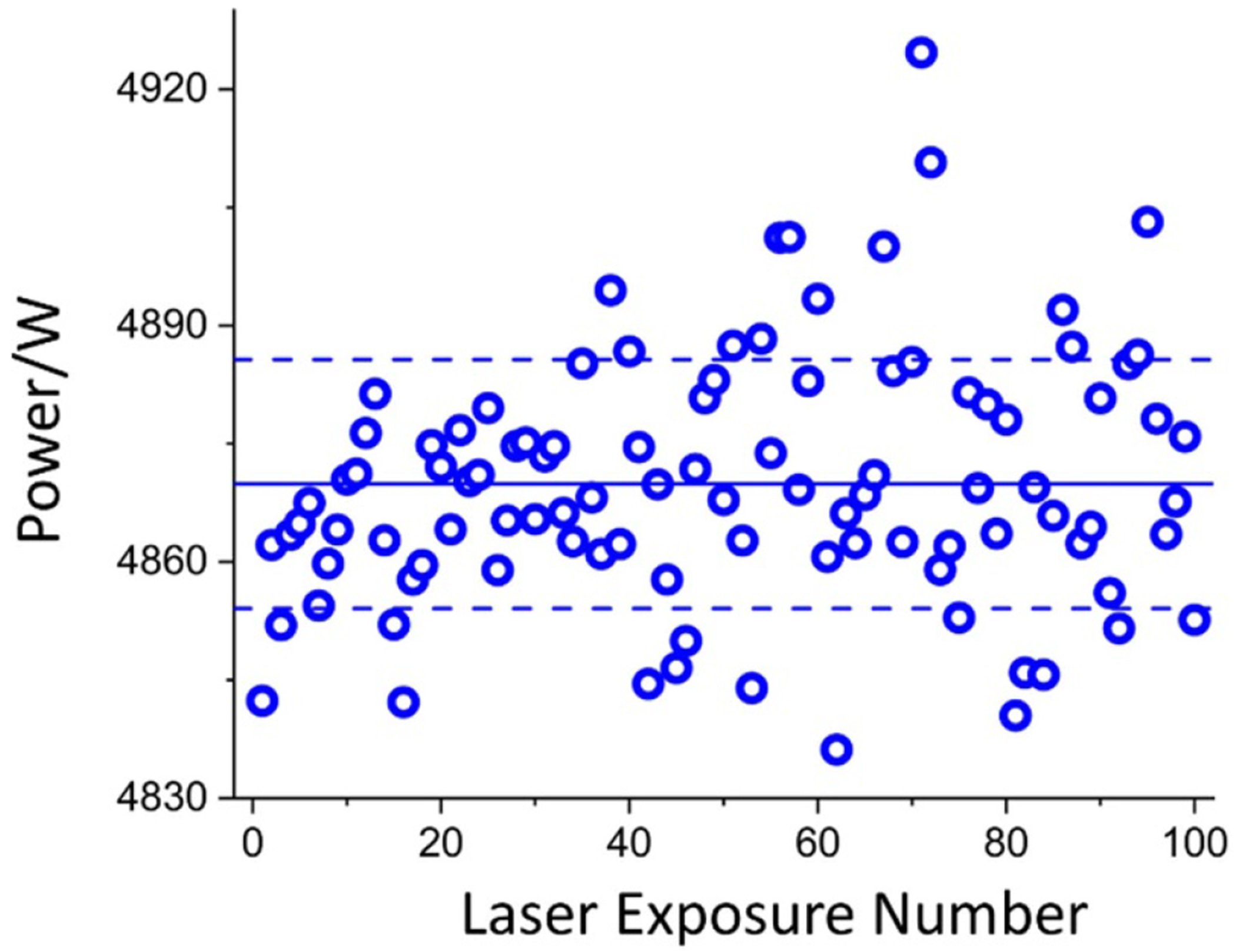
The measured laser power for all 100 laser pulses for 5 kW of nominal applied laser power. The solid line is the mean and the dashed lines are ± one standard deviation.

These regions are shown visually in figure 5(iii). The 10-second laser power ramp before and after region B is excluded from the analysis. The force value of each pulse is converted to a laser power using equation (1). Data at all laser powers were analyzed similarly. Figure 6 shows a point cloud of the measured laser power for all 100 laser pulses of the nominal 5 kW injection with the mean (4869.9 W) given by the solid horizontal line and the standard deviation given by the dashed lines (±15.8 W).

### 3.2. 100 W results

**Figure 7.**
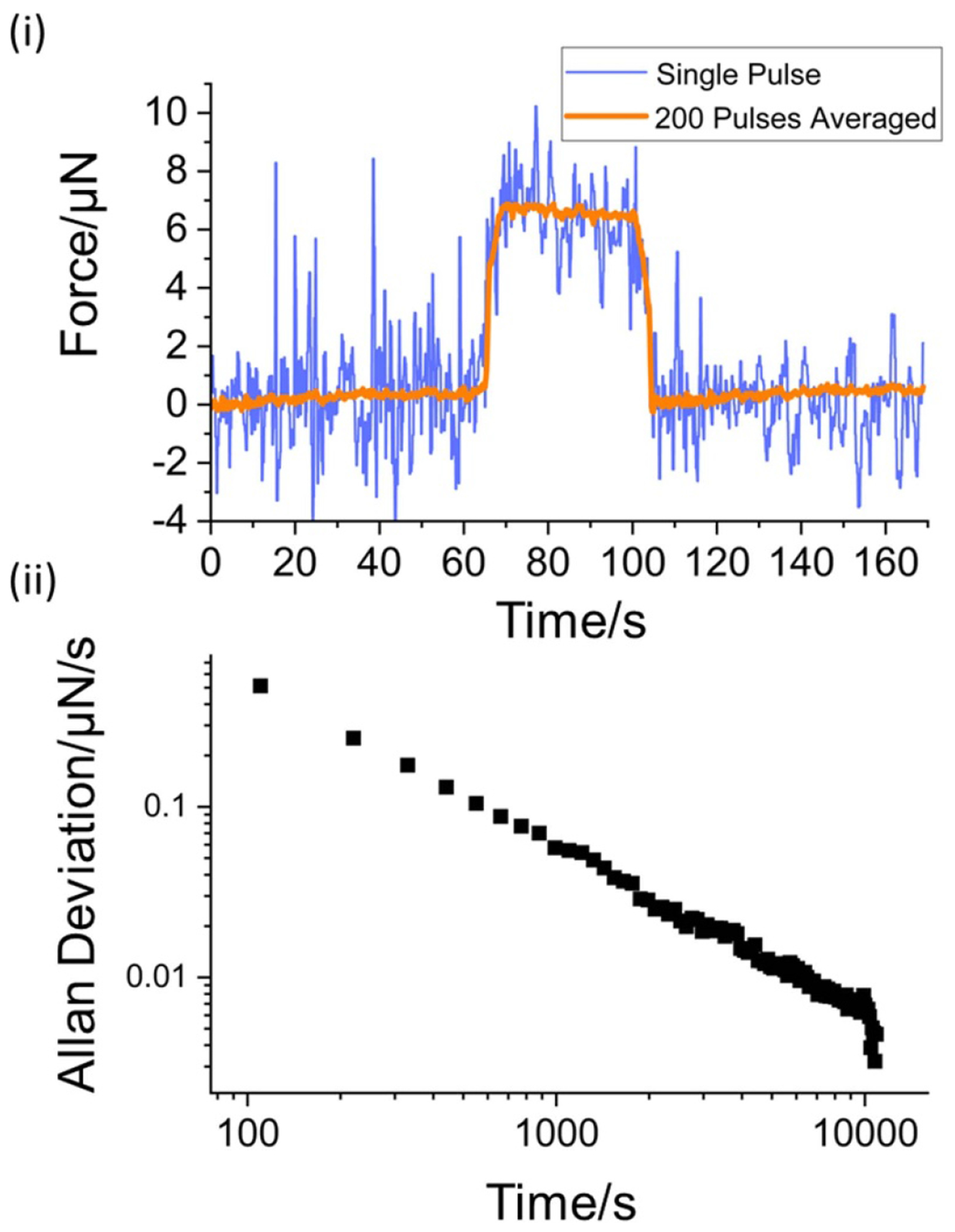
Results of 100 W of nominal applied laser power. The upper plot (i) shows the force as a function of time of a single pulse as well as all 200 pulses averaged. The bottom (ii) shows an Allan deviation plot for all 200 laser pulses.

The dynamic range of the HALO system was assessed by applying 100 W of nominal laser power. This is near the bottom of the stable operating range of the high-power laser. Figure 7(i) shows the force measured by a single laser pulse (blue curve) as well as the average of all 200 pulses (orange curve). The decreasing Allan deviation shown in figure 7(ii) of all 200 laser pulses suggests that the limit of statistical noise reduction through averaging has not yet been met. The measured laser power was 95.06 W ± 0.67 W (0.70%, *k* = 2) with a SDOM of 0.33 W.

At 100 W, which is the lowest stable output of our laser, we achieve a signal-to-noise ratio of almost 300. Based on this, we believe the HALO is potentially capable of resolving optical laser powers near 1 W or below. In order to achieve similar uncertainties at 1 W as we do here (on order of 1%), the following assumptions would need to be made. First, the uncertainty would need to be noise dominated at these low power levels. Second, excepting some additional noise reduction at very low powers, sufficiently long averaging would need to be achieved. By extrapolating the fit in figure 4 down to 1 W, we estimate that this would be achieved in the current HALO system by averaging on the order of 10^5^ laser pulses. Clearly this would be difficult with the laser pulse duty cycle presented here (110 s pulse^−1^). Therefore, measurements near 1 W should include both methods for reducing pulse duty cycle time and additional implementations to reduce statistical noise. We believe both are possible, which will be the subject of future research. Reducing the operating range of HALO to this level would enable validation against two different NIST primary standards spanning the milliwatt [20] to kilowatt regime.

### 3.3. Comparison with thermal primary standard

**Figure 8.**
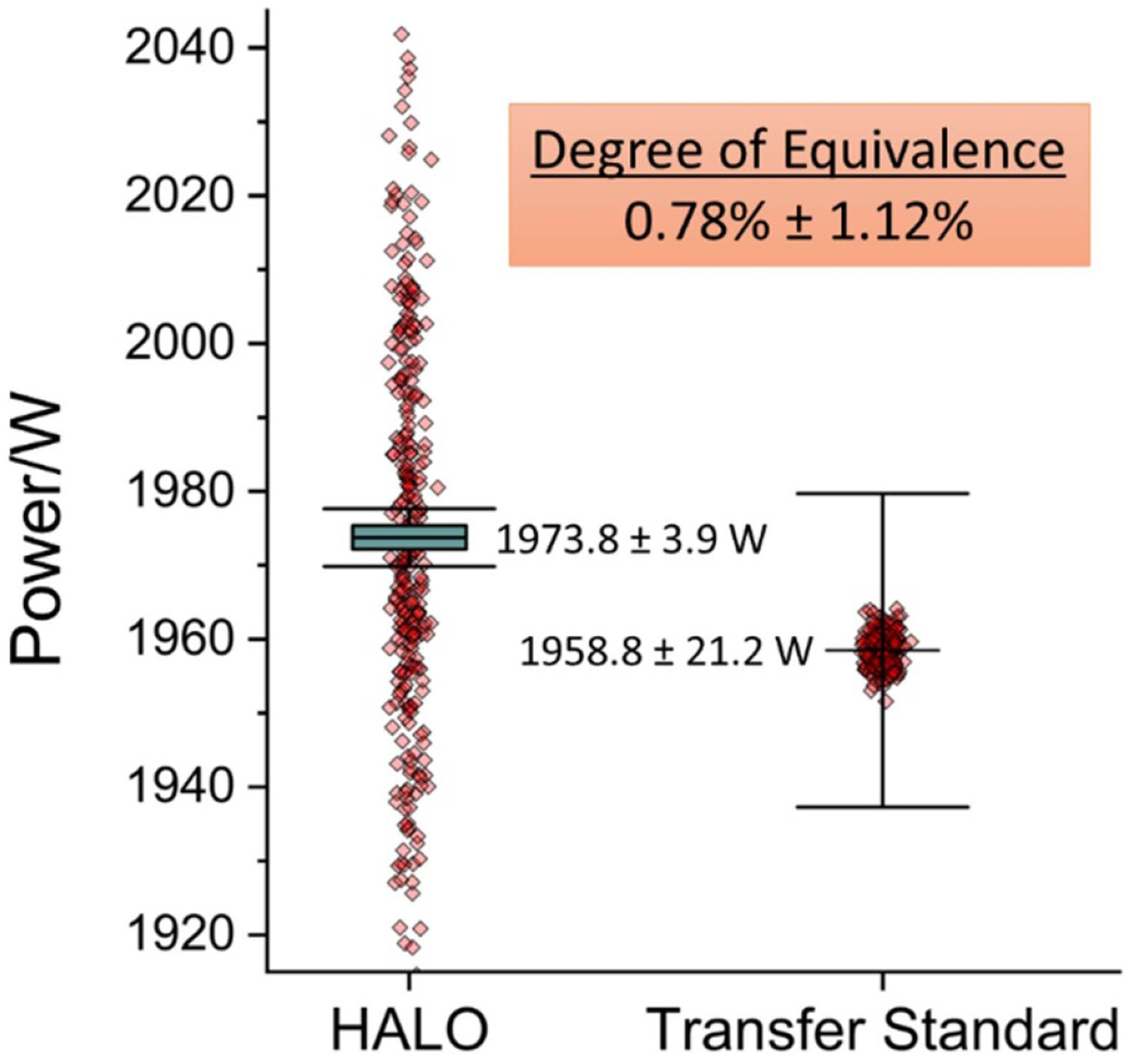
Validation of HALO results against an electrical substitution calorimeter using a transfer standard. The green boxes represent the SDOM of all data points for each measurement with that of the transfer standard too small to see at this scale. The error bars are the expanded uncertainty (*k* = 2).

HALO results were compared against the legacy NIST primary standard, which is an electrical substitution calorimeter [9]. The calorimeter calibrated a thermopile transfer standard that was then used to capture the laser beam exiting the HALO system (see figure 2). The transfer standard was calibrated with 2 kW of nominal applied laser power and had an expanded uncertainty of 1.08%. Three separate 2 kW comparisons were performed at various times over a two-week period. Each of these three measurements involved 100 laser injections. This was done to determine if fluctuating laboratory conditions that occur due to the time of day or day of the week affected the measurement result. These three different measurements agreed very well with each other with a standard deviation of 0.03%. Due to this excellent agreement, the results of all 300 pulses are shown as point clouds in figure 8.

The degree of equivalence, as calculated in [41], of the mean transfer standard and HALO measurements (0.78%) is less than its uncertainty (1.12%) meaning that the two primary standards are in agreement. The mean measurement and absolute expanded uncertainty values (*k* = 2) are shown in figure 8. These are 1973.8 W ± 3.9 W and 1958.8 W ± 21.2 W for HALO and the transfer standard, respectively. The green boxes overlaying the datapoints are the SDOM for each measurement, with the transfer standard SDOM being too small to see at this scale (0.12 W).

**Figure 9.**
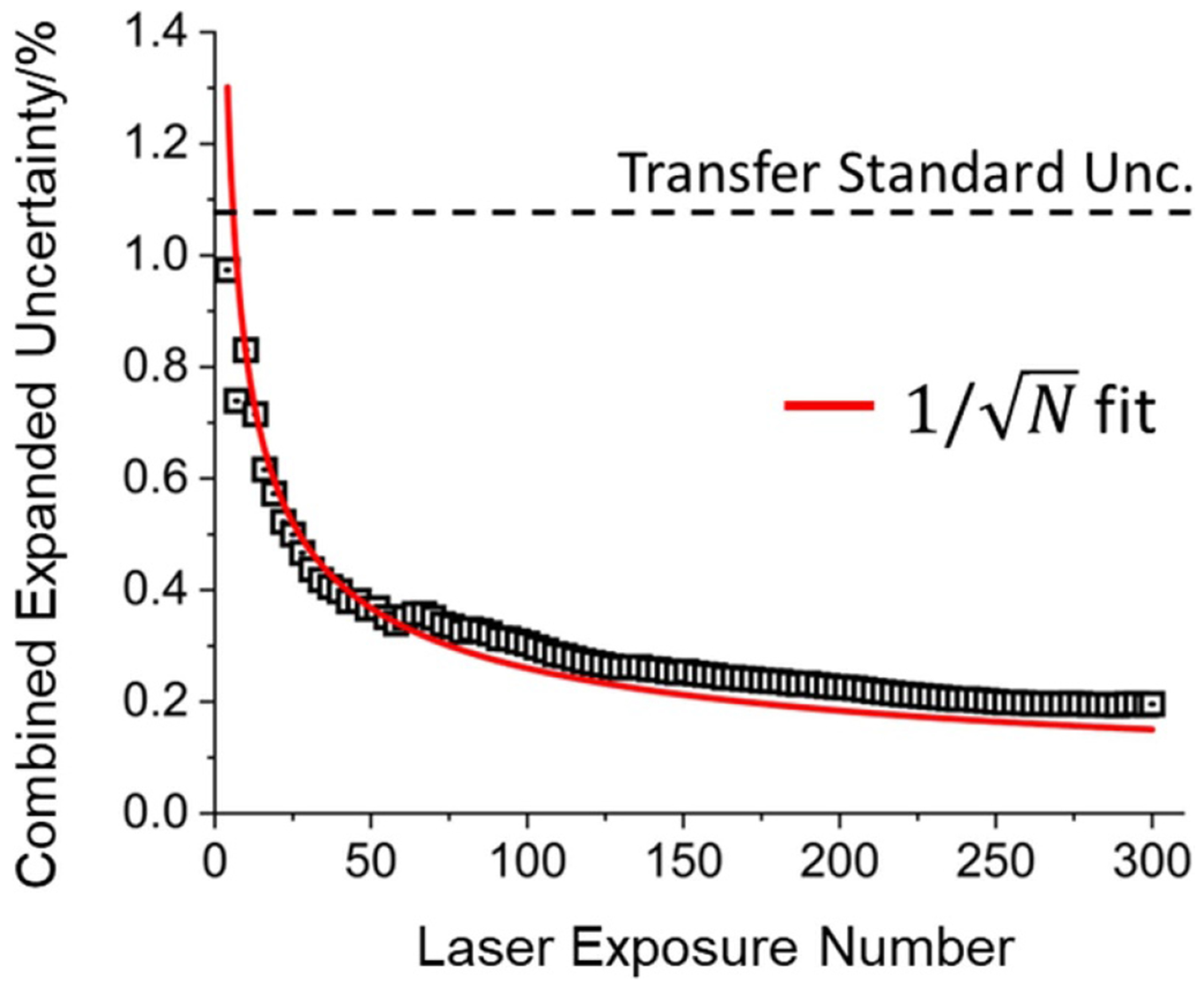
The combined expanded uncertainty of 2 kW HALO data versus the number of laser pulses used for the validation experiment. The red curve is a fit to an inverse square root function.

It is evident from figure 8 that the HALO has a much larger distribution of values than the transfer standard. It is also true that the expanded uncertainty of the HALO is an order of magnitude lower than the transfer standard. This is possible because the systematic uncertainties of the HALO are much lower than those of the electrical substitution calorimeter used to calibrate the transfer standard and the remaining statistical uncertainty can be reduced by averaging. To illustrate that the HALO data are normally distributed, figure 9 shows the expanded uncertainty as a function of laser exposures, which demonstrates an inverse square root dependency. As discussed previously with respect to table 1, this plot will eventually asymptote near 0.1% due to the alignment uncertainty. Although averaging is necessary to achieve the lowest uncertainties with HALO, only a modest number of laser pulses (~5) are required to achieve lower uncertainties at 2 kW than the transfer standard (horizontal dashed line) traceable to the primary standard calorimeter.

## 4. Conclusions

A primary force standard electrostatic force balance was incorporated into our HALO primary optical laser power standard as a practical realization of the watt through Planck’s constant using radiation pressure. The HALO has been used to make the most accurate multi-kW continuous wave laser power measurements ever performed. Measurements of 5 kW nominal injected laser power had an expanded uncertainty of 0.12% (*k* = 2). This also represents the lowest uncertainty radiation pressure-based laser power measurement at any power level. The accuracy of these measurements improved upon previous HALO measurements by roughly a factor of two primarily due to improvements in the alignment uncertainty and improved noise performance from the EFB. From HALO measurements at 100 W, we estimate the low power limit of HALO to be 1 W. HALO measurements were validated against the NIST thermal primary standard power meter with a degree of equivalence of 0.78%, which is less than the combined uncertainty of the two power meters (1.12%).

## Figures and Tables

**Table 1.** Uncertainty budget for HALO measurements.

Uncertainty Component	Type	Relative Uncertainty	Nominal Applied Power			
			0.1 kW	1 kW	2 kW	5 kW
Optical Alignment	B	0.049 %				
Noise (SDOM)	A		0.345 %	0.069 %	0.061 %	0.032 %
Collimator	B	0.016 %				
Corner Loading	B	0.011 %				
Surface Potential	B		0.0024 %	0.0030 %	0.0033 %	0.0032 %
Capacitance Gradient	A	0.0023 %				
Voltage	B	0.0016 %				
Stray Capacitance	B	0.00025 %				
Capacitor Alignment	B	0.00015 %				
Reflectance	B	0.0006 %				
Capacitor Motion	B	0.0001 %				
Transfer of Length	B	0.00005 %				
Expanded Uncertainty (*k* = 2)			0.70 %	0.17 %	0.16 %	0.12 %

## Data Availability

The data that support the findings of this study are available upon request from the author.
